# A giant Jurassic hunter: the earliest dipluran and its implications for hexapod body pattern evolution

**DOI:** 10.1098/rspb.2026.1207

**Published:** 2026-10-07

**Authors:** Yehao Wang, Chenyang Cai, Alba Sánchez-García, Alberto Sendra, Diying Huang

**Affiliations:** ^1^ State Key Laboratory of Palaeobiology and Stratigraphy, Nanjing Institute of Geology and Palaeontology, Chinese Academy of Sciences, Nanjing 210008, People's Republic of China; ^2^ University of Chinese Academy of Sciences, Beijing 100049, People's Republic of China; ^3^ Departament de Botànica i Geologia, Facultat de Ciències Biològiques, Universitat de València, Burjassot 46100, Spain; ^4^ Departament de Didàctica de les Ciències Experimentals i Socials, Facultat de Magisteri, Universitat de València, València 46022, Spain; ^5^ Nanjing College, University of Chinese Academy of Sciences, Nanjing 211135, People's Republic of China

**Keywords:** diplura, hexapod evolution, mouthpart, entognathy, grasping apparatus

## Abstract

Hexapoda comprises the hyperdiverse insects and three cryptic, early-diverging lineages, Protura, Collembola, and Diplura, that are key to understanding the hexapod body pattern but remain extremely rare as fossils. Here, we report the earliest dipluran, *Sinojapyx giganteus* gen. et sp. nov., from the Middle Jurassic Daohugou Biota (approx. 165 Mya). Its ectognathous mouthparts challenge the conventional view that all non‑insect hexapods are entognathous, providing solid evidence that entognathy evolved independently at least three times. The pincer‑like cerci of *Sinojapyx* are symmetrical and lack denticles, representing a transitional state preceding the sophisticated predatory appendages of extant japygoids. Exceeding 5 cm in body length and equipped with robust mandibles and pincer-like cerci, this giant soil dweller occupied the apex of the soil food web in the Jurassic, filling a niche typically held by scorpions or centipedes today. This discovery not only bridges a major gap in the dipluran fossil record but also offers critical insights into the ancestral body pattern of non‑insect hexapods and soil trophic networks in the Jurassic.

## 1. Introduction

Hexapods (six-legged arthropods) are the most successful terrestrial metazoan lineages, encompassing the extraordinarily diverse insects as well as three relatively species-poor groups: Protura, Collembola, and Diplura [1]. Although insects dominate terrestrial biodiversity, the non-insect hexapods comprise less than 1% of described animal diversity [2–4]. However, these lineages occupy a key phylogenetic position for reconstructing the origin and early evolution of the hexapod body pattern [1]. Their ancient origins and long-standing adaptation to soil environments have resulted in numerous morphological autapomorphies and homoplasies, complicating the resolution of phylogenetic relationships among the four hexapod groups [5]. The ‘Entognatha’ hypothesis, first proposed by von Stummer-Traunfels [6] for Collembola and Diplura, is based on the unique mouthparts of these groups, which are recessed within a gnathal pouch of the head capsule [7]. This concept was later extended to include Protura and was widely accepted for some time [8], although recent phylogenomic studies have challenged it [1,9].

Diplura, or two-pronged bristletails, are considered closely related to insects owing to shared microstructural features in their ovaries and sperm, making them a key group for understanding the early evolution of insects [10]. To date, approximately 1000 species have been described, distributed across 10 families and three superfamilies [4]. Diplurans inhabit a variety of ecosystems, from subterranean habitats like caves to the surface layers of organic litter, where they play a crucial role in maintaining soil ecological balance [11,12]. One of the most distinguishing characteristics of diplurans is the state of highly specialized cerci at the end of the abdomen. Campodeoidea, distinguished by their elongated, filamentous cerci, exhibit adaptations for sensory functions, whereas Projapygoidea possess short, segmented cerci equipped with expelling glands, and Japygoidea are characterized by pincer-like cerci, which are specialized adaptations enhancing their predatory capabilities [13]. Some large species within the japygoid families Heterojapygidae and Dinjapygidae occupy the apex of the invertebrate trophic pyramid, filling a niche typically occupied by large invertebrates such as scorpions and chilopods [4,14]. Despite the ecological significance of pincer-like cerci, limited behavioural data constrain our understanding of their functional morphology, adaptive significance, and the ecological pressures that have shaped their forms [15].

The scarcity of non-insect hexapod fossils presents a significant challenge to understanding the origin and evolution of hexapods. To our knowledge, no proturan fossils have been discovered to date. The Early Devonian fossils of Collembola and insect fragments represent the earliest known records of Hexapoda, offering a glimpse into early terrestrial ecosystems [16–18]. However, owing to poor preservation and their morphological resemblance to extant groups, these fossils offer limited evolutionary insights. A significant discovery is the Pennsylvanian *Testajapyx thomasi* from Mazon Creek, Illinois, North America [19], interpreted as a stem group of Japygoidea based on its prominent compound eyes and pincer-like cerci [20]. However, Bitsch [21] noted that these cerci were associated with the eighth abdominal segment (in contrast with the tenth segment in extant Japygoidea) and argued that the presence of terminal pincer-like cerci alone is insufficient to establish its affinity with Diplura. Consequently, its phylogenetic placement within the hexapod lineage remains unresolved [22]. The first unequivocal dipluran fossil, *Ferrojapyx vivax*, is reported from the Early Cretaceous Crato Formation in Brazil [23]. Moreover, since the Cretaceous, ambers and unique deposits like the Onyx marble of Arizona have provided exceptional preservation of numerous non-insect hexapods [24–30]. Apart from the springtails *Rhyniella praecursor* (Devonian) [16] and *Permobrya mirabilis* (Permian) [31], a substantial gap in the fossil record of non-insect hexapods exists between the Devonian and Cretaceous.

In this study, we report the earliest known dipluran, represented by a single specimen that is exceptionally rare among nearly 300 000 fossils examined from the Daohugou Biota, both in field collections and institutional repositories. The specimen originates from the fossil-bearing layer of the Haifanggou Formation at the Xiayingzi Quarry. This horizon represents the peak of biodiversity within the Daohugou beds, having yielded a diverse assemblage of remarkable insects [32].

## 2. Systematic palaeontology

Superclass: Hexapoda Latreille, 1825 [33]

Class: Diplura Börner, 1904 [34]

Superfamily: Japygoidea Lubbock, 1873 [35]

Family: **Sinojapygidae** fam. nov.

Type genus: ***Sinojapyx*** gen. nov.

Diagnosis. Body size gigantic, exceeding 5 cm (more than twice the length of typical extant Japygoidea, which are usually < 2 cm); head nearly triangular; mouthparts ectognathous, robust mandibles with large teeth and laciniae lacking pectinate laminae; maxillary and labial palps multi-segmented; abdominal segment IX strongly elongated; segment VIII−X not more sclerotized than the rest of abdomen, with urosternite X partly fused to the pleurite; pincer-like cerci equal, devoid of teeth and denticles.

ZooBank LSID: urn:lsid:zoobank.org:act:7D7D3E8D-5852-4293-8642-97D94EAFB6CF

Genus: ***Sinojapyx*** gen. nov.

Type species: ***Sinojapyx giganteus*** sp. nov.

Etymology. The prefix, ‘Sino-,’ refers to China. ‘Japyx’ denotes the type genus of the family Japygidae. The gender is masculine.

Diagnosis. As for family.

ZooBank LSID: urn:lsid:zoobank.org:act:AA194013-51D1-4D56-BF24-EE994CFD388B

***Sinojapyx giganteus*** sp. nov. (figures 1, 2, and 4; supplementary material, figure S1)

**Figure 1.**
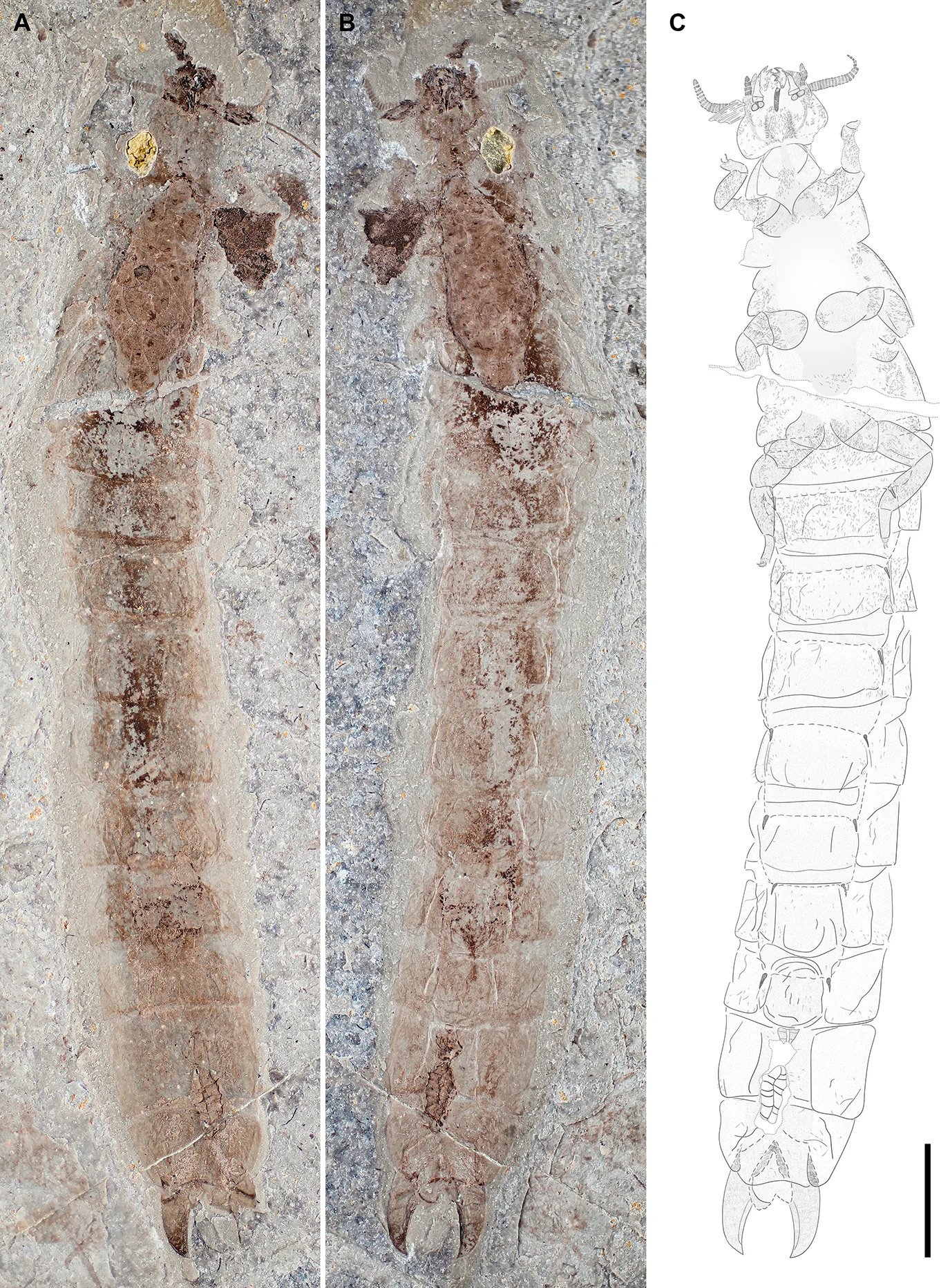
General habitus of *Sinojapyx giganteus* (holotype, NIGP205729) from the Middle Jurassic Daohugou Bed. (A) Counterpart, inner view of dorsal aspect. (B) Part, inner view of ventral aspect. (C) Line drawing based on the part with reference to the counterpart; scale bar, 5 mm.

**Figure 2.**
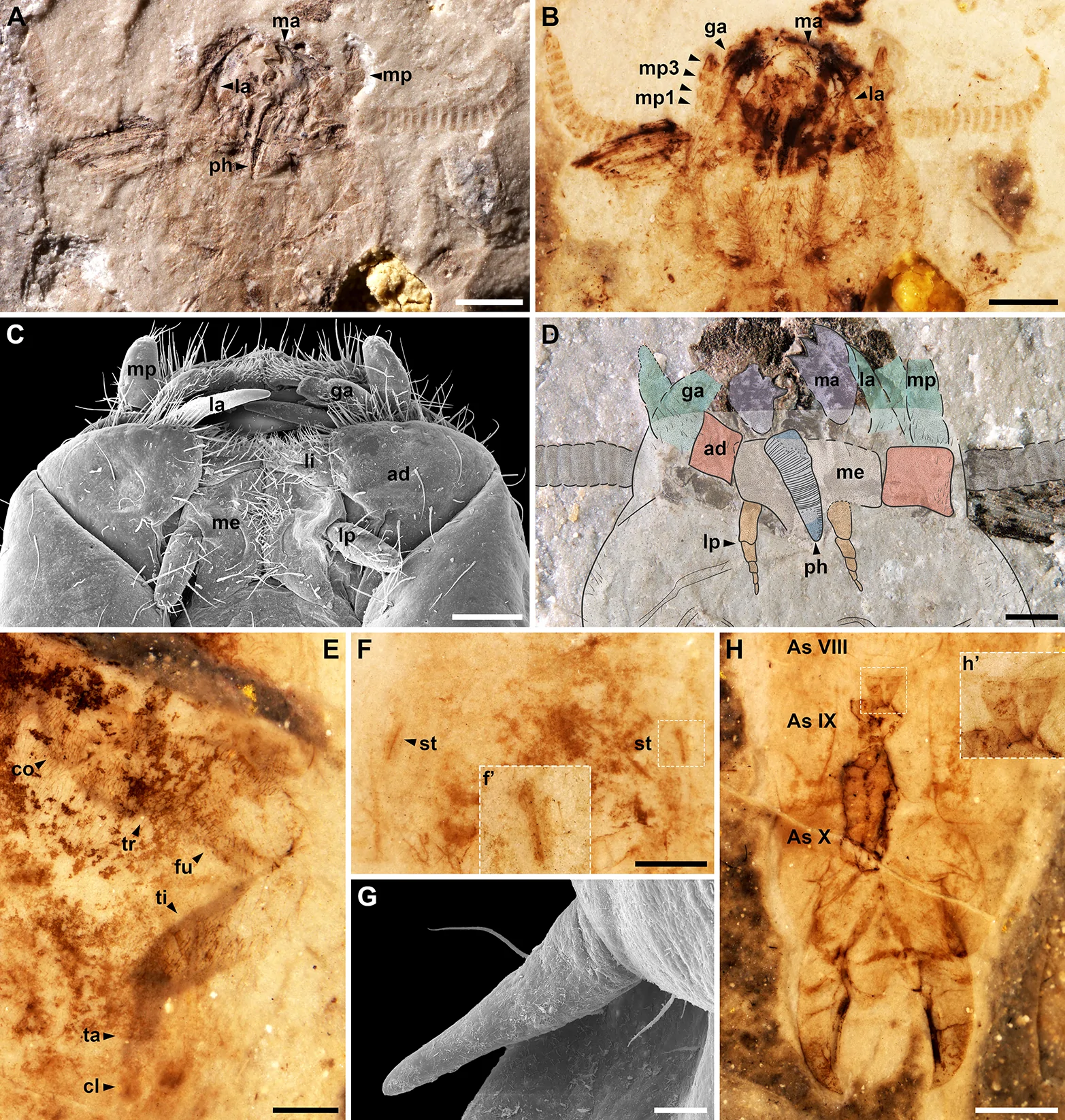
Details of holotype of *Sinojapyx giganteus* and comparison of extant *Heterojapyx* sp. (A and B) Head on the part. (C) Ventral scanning electron microscopy (SEM) view of the mouthparts of *Heterojapyx*. (D) Mouthparts on the counterpart, with line drawings and colour differentiation to highlight individual structures. (E) Right hindleg of part. (F) Styli on abdominal segment V of the part, f’, magnified view of the styli. (G) SEM view of styli in *Heterojapyx*. (H) Abdominal segments IX–X with pincer-like cerci on the part, h′, magnified view of the genital appendages. (B, E, F, and H) are photographed under ethanol. Abbreviations: ad, admentum; As VIII, IX, X, abdominal segments VIII, IX, X; cl, claw; co, coxa; fu, femur; ga, galea; la, lacinia; li, ligula; lp, labial palp; ma, mandible; me, mentum; mp, maxillary palp; ph, pharynx; st, styli; ta, tarsus; ti, tibia; tr, trochanter. Scale bars: (A, B, E, F) 1000 µm; (C) 250 µm; (D) 500 µm; (G) 50 µm; and (H) 2000 µm.

Material. Holotype, NIGP205729a, b, deposited in Nanjing Institute of Geology and Palaeontology, an adult male, from the Middle Jurassic Daohugou Bed (approx. 165 Mya), Inner Mongolia, China.

Etymology. The specific epithet is derived from the Latin word ‘gigante’ which means ‘giant.’ The gender is masculine.

Diagnosis. As for the genus.

Description. Specific descriptions are provided in the supplementary material.

Remarks. Specific remarks are provided in the supplementary material.

ZooBank LSID: urn:lsid:zoobank.org:act:5D95C419-C145-4171-B0D0-B8E05504DE61

## 3. Discussion

### (a) Independent origins of entognathy in Hexapoda

The non-insect hexapod lineages (Protura, Collembola and Diplura) emerged early, yet their body patterns exhibit significant specialization, such as segment reduction and eye degeneration. Given the limited fossil record, our understanding of the ground body pattern of Hexapoda remains elusive. A key issue lies in the ‘Entognatha’ hypothesis [36,37], which is defined by mandibles and maxillae enclosed within a gnathal pouch formed by folds of the oral cavity (figure 2C) [38–42]. However, the monophyly of Entognatha sensu Hennig [8] has been repeatedly questioned based on both anatomical and molecular evidence [9,19,41].

The giant Jurassic *Sinojapyx giganteus* displays many diagnostic features of Diplura, including a pair of multi-segmented antennae (figure 2A,B), three thoracic segments with each bearing a pair of five-segmented legs (figures 1 and 2E; supplementary material, figures S1 and S2G‒S2I), 10 abdominal segments with a pair of conical styli on urosterites I to VII (figures 1 and 2F,f’,G; supplementary material, figure S1), and a pair of cerci (figure 2H) [43]. In extant diplurans, the admentum, derived from the labial coxa, is well-developed and positioned anterolaterally, facilitating a wide oral opening that enhances biting efficiency (figure 2C) [38]. By contrast, the admentum of *S. giganteus* extends only to the base of the mandibles and the middle portion of the laciniae, coupled with the mentum and maxillary palps located further posteriorly (supplementary material, figure S2D‒S2F). These features suggest that *S. giganteus* retained an ectognathous condition, with the mandibles and laciniae partially exposed (figure 2D), unlike extant diplurans, in which the mandibles are fully enclosed (figure 2C).

As a specialized group of terrestrial crustaceans [44], hexapods are hypothesized to have inherited an ectognathous mouthpart configuration as the ancestral condition [41]. However, with the exception of Insecta, the three other early-diverging hexapod lineages, Protura, Collembola and Diplura, all exhibit entognathous mouthpart structures [6,8]. Morphological studies of the extant representatives of these three lineages have revealed that the arrangement of mouthparts differs sufficiently to suggest that entognathy evolved at least twice independently, with Diplura displaying a distinct condition from that of Protura and Collembola [41]. The earliest known Devonian hexapod fossil, *Rhyniella praecursor* (Collembola), already exhibits highly specialized entognathous features remarkably similar to those of its modern relatives [45]. However, Jurassic *S*. *giganteus* still exhibits an ectognathous configuration. Therefore, our discovery not only provides solid evidence for the ectognathous configuration as the ancestral state, but also supports the independent origins of entognathy in the non-insect hexapod lineages. Recent phylogenomic studies have challenged traditional views of hexapod evolution, identifying Protura as the earliest-diverging hexapod lineage and placing Collembola and Diplura (i.e. Entognatha, sensu [6]) as the sister group to Insecta [1]. Based on this updated phylogenetic framework and new fossil evidence from this study, we propose that entognathy evolved independently at least three times within the non-insect hexapod lineages.

### (b) Phylogeny resolves *Sinojapyx* as a stem japygoid

The specialized pincer-like morphology of cerci in *Sinojapyx* suggests its close relationship with Japygoidea. Extant japygoids typically display narrowed and highly sclerotized abdominal segments VIII‒X [46,47]. By contrast, these segments in *Sinojapyx* do not show a more pronounced degree of sclerotization compared with the preceding abdominal segments (figure 1; supplementary material, figures S1, S2K‒S2L). Another distinguishing characteristic is the unreduced abdominal segment IX (figures 1 and 2H; supplementary material, figure S1), which resembles that of extant campodeoids and projapygoids; in contrast, this segment has been reduced to the shortest abdominal segment in japygoids since at least the Cretaceous [23]. Moreover, *Sinojapyx* exhibits multi-segmented labial palps, maxillary palps, and genital appendages (figure 2A,B,D,h’; supplementary material, figure S2A–S2F), a plesiomorphic character corroborated by the Paleozoic archaeognathan *Dasyleptus* species and their extant relatives [19,48]. Overall, *Sinojapyx* presents a strikingly mosaic combination of traits: although it possesses the diagnostic features of Japygoidea, it retains remarkable primitive characteristics that are absent in all extant Diplura (supplementary material).

To clarify the precise systematic position of *S. giganteus*, we conducted a morphological phylogenetic analysis, which consistently recovered it as a stem-group representative of Japygoidea (figure 3A). We also incorporated the Cretaceous *F. vivax* into our analyses, but its systematic position remained unresolved in the absence of *S. giganteus* (supplementary material, figures S3B, S4B, and S5B). By contrast, when *S. giganteus* was included, *F. vivax* was recovered as the sister group to all extant japygoids (figure 3A; supplementary material, figures S3A, S4A, and S5A), highlighting the evolutionary significance of *S. giganteus* as a transitional form.

**Figure 3.**
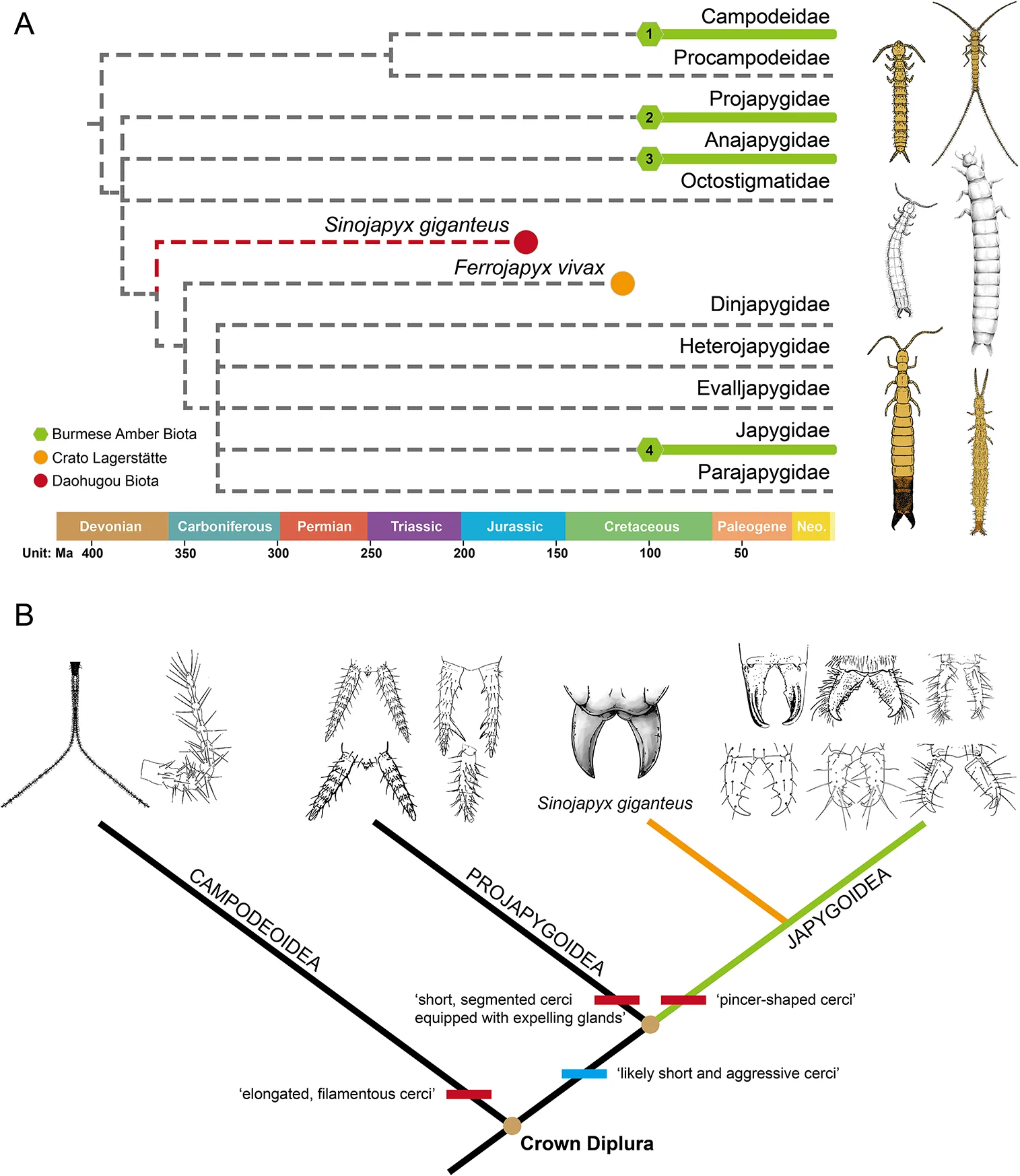
Phylogenetic and evolutionary context of Diplura. (A) Phylogeny of Diplura showing the phylogenetic placement of *Sinojapyx giganteus* and *Ferrojapyx vivax*. Red and yellow circles indicate extinct species. The thick green bar represents the currently known temporal range of each family, with the following taxa indicated: (1) Lepidocampinae species (supplementary material, figure S6); (2) *Electroprojapyx alchemicus* [27]; (3) *Eoanajapyx yinae* [30]; and (4) *Cretojapyx huangi* [29]. Illustrations of diplurans (from top to bottom: Campodeidae, Projapygidae, *F. vivax*, *S*. *giganteus*, Heterojapygidae, and Parajapygidae) are drawn by Jie Sun or adapted from Sendra [43] and Wilson & Martill [23]. Currently, no temporal framework is available for the evolution of diplurans, and the dashed lines therefore represent inferred divergence times. (B) Evolutionary context of the cerci in Diplura. Representatives of extant cerci were redrawn based on *Procampodea* [49] and *Lepidocampa* [50] (Campodeoidea); *Anajapyx*, *Symphylurinus* [51], and *Octostigma* [52] (Projapygoidea); *Heterojapyx* [53], *Dinjapyx* [54], *Provalljapyx* [55], *Kohjapyx* [56], *Evalljapyx* [57], and *Parajapyx* [58] (Japygoidea). Red bars indicate the cercus types of the three superfamilies, and blue bars indicate inferred cercus innovations. The yellow branch indicates *S. giganteus*, a stem-group member of Japygoidea identified in this study, with its cercal reconstruction illustrated by Chao Tan.

### (c) Evolutionary transformation of dipluran cerci towards a grasping apparatus

Cercal grasping is a crucial behaviour among various representatives of Hexapoda [59,60]. For instance, earwigs have developed diverse pincer-like cerci, akin to those of japygoids, which perform multiple functions such as defence, predation, wing unfolding, and sexual interactions [60]. Kosaroff [61] and Schaller [62] examined the feeding behaviour of japygoids and observed that they typically grasp and consume prey immediately upon encountering it. If the prey resisted, japygids would curl their abdomen upwards and forwards, utilizing their cerci to secure the prey [61‒63]. Additionally, when prey was concealed in soil crevices, they would insert their abdomen into the crevice and extract the prey using their cerci [62]. Thus, cerci play a pivotal role in the predatory system of Diplura, exhibiting significant morphological diversity and housing mechano- and chemoreceptors that assist in prey detection and handling [15].

By contrast, the cerci of *S. giganteus* are highly simplistic (equal and lacking teeth and denticles; figure 2H). Some extant japygid genera, such as *Rectojapyx* and *Psalidojapyx* [64,65], also lack teeth and possess only a few proximal denticles on their equal cerci. These features are considered part of the ancestral ground pattern [66–68] and represent the morphology observed during the first juvenile stages, where the inner margin of both cerci is smooth [63,69]. The complete smooth inner margin characteristic of *S. giganteus* is absent in modern japygoids but has been documented in ancient species. For example, the Early Cretaceous japygoid *F. vivax* from the Crato Formation in Brazil exhibits similar cerci without distinct denticles [23]. Besides, the sclerotization of abdominal segment X facilitates the attachment of numerous muscles associated with cercal movement (supplementary material, figure S2J). However, in *S. giganteus*, the relatively soft and incompletely fused condition of segment X probably provided limited mechanical support for cercal operation. Nonetheless, the presence of its sharp margins and central groove may have contributed to a cutting function during predation. Furthermore, the specializations of the posterior three abdominal segments in extant japygoids may function analogously to the ‘wasp waist’ in Apocrita, enhancing flexibility for manipulating long ovipositors at the abdomen's terminus [7]. By contrast, the broad and elongated tail region of *S. giganteus* suggests reduced manoeuverability in cercal control compared with its extant counterparts.

Therefore, the cerci of *S. giganteus* probably represent an ancestral state in the evolution of this grasping apparatus, reflecting the body pattern of the ancestral japygoid (figure 3B). By mapping cercal phenotypes onto the phylogenetic framework of Diplura, we tentatively infer that dipluran cerci probably underwent a major evolutionary innovation from elongate, sensory appendages to shortened cerci specialized for prey capture and other offensive or defensive functions (figure 3B). Extant Campodeoidea are primarily omnivorous generalists, feeding on a wide range of resources, including fresh roots, fungal hyphae and spores, decaying organic matter, and even minute invertebrates [4]. By contrast, although occasional omnivorous and herbivorous habits have been reported in some extant Projapygoidea and Japygoidea [52,70,71], most species in these groups are predominantly carnivorous [4]. Accordingly, this trophic shift may have been one of the principal drivers underlying the diversification of cercal morphology. Furthermore, the transition from simplistic, smooth cerci in *Sinojapyx* to the complex, denticulated predatory apparatus of extant japygoids probably reflects the outcome of an evolutionary arms race between predator and prey over their long evolutionary history.

### (d) *Sinojapyx* was a giant top predator

The rich palaeofloral assemblage of the Daohugou area indicates a forested ecosystem with moist conditions under a warm‑temperate climate [72]. Extant diplurans are highly sensitive to environmental parameters, depending strongly on high humidity and moderate temperatures [11]. They reach their greatest diversity in temperate forests with nutrient‑rich soils [4]. The palaeoenvironment of the Daohugou region closely matches the ecological requirements of extant diplurans, providing optimal conditions for their survival.

In well-stratified soils, modern diplurans actively colonize all soil horizons, with larger japygoids predominantly occurring in the organic layer and topsoil [4]. By contrast, smaller species inhabit narrow crevices in deeper subsoil and exhibit adaptations such as reduced body length (typically < 2 mm) and shorter appendages [4]. *S*. *giganteus* exceeds 5 cm in body length, making it larger than about 98% of extant dipluran species [4]. Among living forms, only the giant dinjapygids and heterojapygids reach comparable sizes; they thrive as apex invertebrate predators beneath rocks and even occupy a niche typically filled by large invertebrates such as scorpions and chilopods [4,14].

The mandible of *S. giganteus* is robust. Recent japygoids possess slender mandibles with several teeth on the distal portion [38,73]. By contrast, *S. giganteus* bears a strongly curved mandible equipped with three sturdy, sharp teeth that occupy the anterior two-thirds (figure  2A; supplementary material, figure S2A–S2C). In extant japygoids, the lacinial tooth is strongly hooked, which restricts the gape angle and limits the size of food particles that can be ingested [70]. The inner margin of this tooth, however, carries pectinate laminae [70,73–75] that serve a wiping function and facilitate the uptake of soft tissues and liquids [70]. By comparison, the lacinial tooth of *S. giganteus* is only slightly curved and lacks such laminae (figure  2A ,B; supplementary material, figure S2A–S2C), implying a wider gape angle than in extant relatives and the ability to ingest larger food items. Thus, the mouthparts of *S. giganteus* conferred greater tearing and chewing capabilities than those of modern japygoids, enhancing its predatory efficiency. Furthermore, although its pincer-like cerci remain in a relatively primitive state, they are already very robust. Extant giant heterojapygids can conceal themselves in soil galleries with only their cerci exposed at the surface, ready to grasp passing prey [76]. Given its similarly large body size and short, robust legs, *S*. *giganteus* was probably not a speed‑dependent predator. Instead, we propose that it probably functioned as an ambush hunter in Jurassic soils, using its pincer‑like cerci to opportunistically seize passing prey.

The Daohugou biota represents one of the most biodiverse Lagerstätten, comprising a large number of edaphic taxa [77]. Most of these are omnivorous (e.g. jumping bristletails, cockroaches [32]). Some predatory beetles (e.g. caraboids) and spiders are also present but are relatively small [32,78]. Moreover, typical top predators of the soil, such as scorpions or centipedes, have not yet been recorded from this biota. Therefore, based on current evidence, the giant *S. giganteus* is among the apex predators in the soil ecosystem of the Daohugou biota (figure 4).

**Figure 4.**
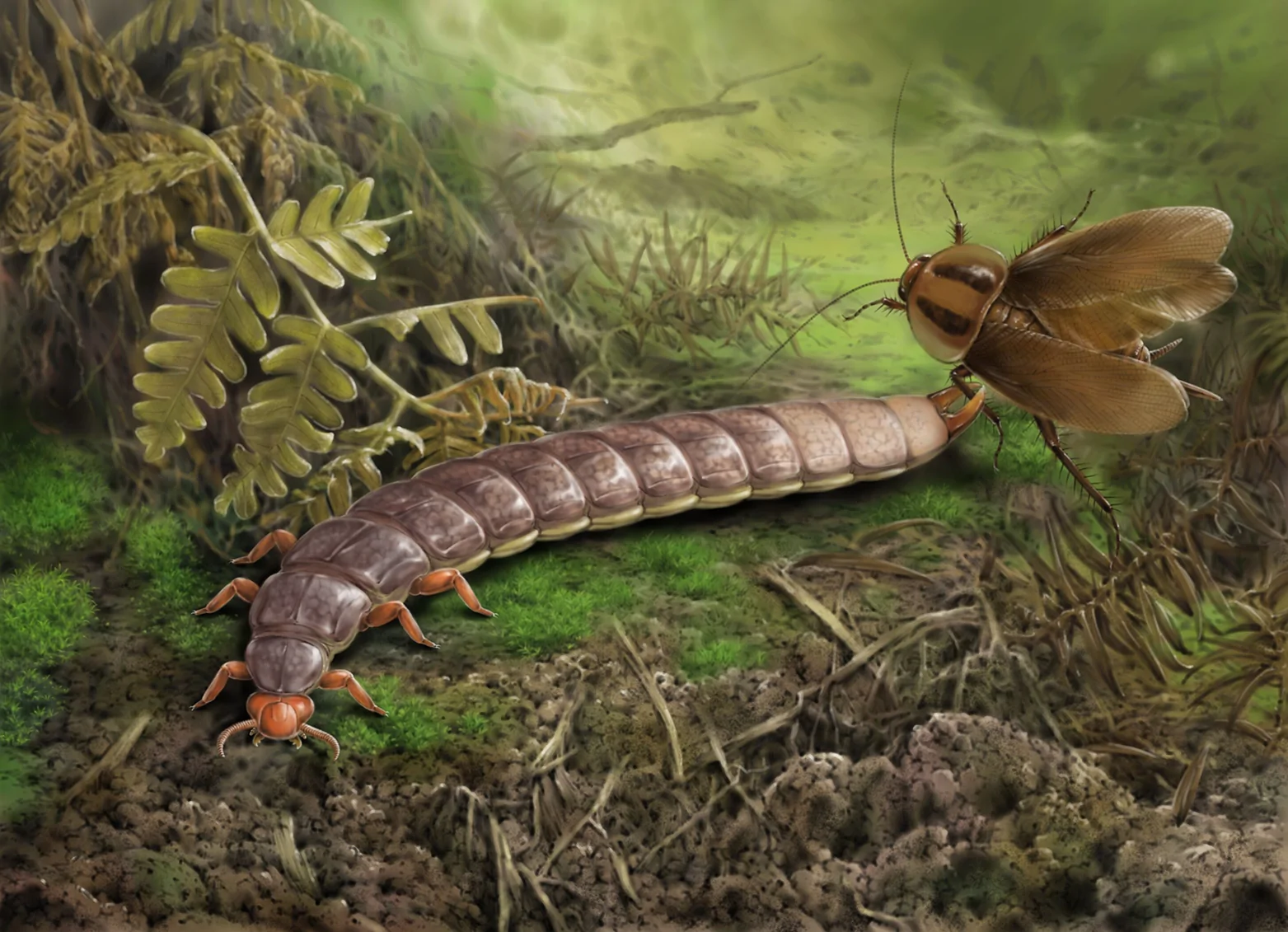
Palaeoecological reconstruction of *Sinojapyx giganteus,* illustrated by Jie Sun.

## 4. Material and methods

### (a) Material

The fossil specimen examined in this study is the only dipluran recovered to date among the hundreds of thousands of fossils from the Daohugou biota (Haifanggou Formation, Daohugou, Ningcheng County, Inner Mongolia, northeastern China) collected by our research group. The specimen is exceptionally well preserved and split into part and counterpart. It was carefully prepared using a sharp knife and is deposited in the Nanjing Institute of Geology and Palaeontology, Chinese Academy of Sciences.

### (b) Fossil imaging

General habitus of *S. giganteus* was captured using a Canon 5D camera paired with a 100 mm macro lens. Detailed brightfield images were captured with a Zeiss Stereo Discovery V16 stereomicroscope, with 70% alcohol applied to enhance contrast. Images of the extant species were obtained via scanning electron microscopy (SEM) following gradient dehydration and critical point drying in Jiangsu Academy of Agricultural Sciences. Where necessary, photographs were stacked using Helicon Focus 7.0.2 and adjusted for brightness and contrast in Adobe Photoshop 2020. The phylogenetic tree was finalized and edited for aesthetical purposes in Adobe Illustrator 2020. Imaging equipment are supported by the Nanjing Institute of Geology and Palaeontology, Chinese Academy of Sciences.

### (c) Terminology

The morphological terminology follows Pagés [47,64], Smith [55], and Blanke & Machida [38].

### (d) Phylogenetic analyses

We conducted a morphology-based phylogenetic analysis to evaluate the systematic placement of the new fossil family. Owing to the scarcity of family-level diagnostic characters applicable to extant diplurans, as well as limitations in specimen availability and the current state of taxonomic knowledge, no dedicated morphological data matrix for extant Diplura have been available to date. In this study, we therefore constructed a matrix comprising 17 morphological characters, covering 10 extant families and two fossil taxa, *S. giganteus* and *F. vivax* (supplementary material). Two control matrices were established to assess the impact of including or excluding Sinojapygidae (supplementary material). Phylogenetic analyses were conducted using a maximum parsimony approach, based on the published one-step TNT script *guoyi.run* [79,80]. Implied weighting was applied, with the concavity constant set to 12 following the recommendations of Goloboff *et al.* [81] and Smith [82]. Consensus trees were generated using three strategies: strict consensus (*str*), majority rule (*mjr*) consensus, and semi-strict (*hlf*) consensus. Node support was assessed using three resampling methods: jackknife, bootstrap, and symmetric resampling. The resulting tree was visualized using FigTree v1.4.4 (https://github.com/rambaut/figtree) and graphically edited with Adobe Illustrator 2020.

## Supplementary Material



## Data Availability

Phylogenetic datasets and supplementary figures are available in the supplementary material. Supplementary material is available online [83].
