# Supplementary material for: A giant Jurassic hunter: the earliest dipluran and its implications for hexapod body pattern evolution
Source: Proc Biol Sci. 2026 Oct 7;293(2080):20261207. doi: 10.1098/rspb.2026.1207 (PMC13641902; doi:10.1098/rspb.2026.1207)
Supplement: Supplementary file 1 [file rspb.2026.1207.zip › rspb20261207_si_001.docx]

**Supplementary Material for**

A giant Jurassic hunter: the earliest dipluran and its implications for hexapod body plan evolution

Yehao Wang, Chenyang Cai, Alba Sánchez-García, Alberto Sendra, Diying Huang

Proceedings of the Royal Society B: Biological Sciences

DOI: 10.1098/rspb.2026.1207

**Contents of this file:**

Description

Remarks

Morphological characters and character states used in phylogenetic analyses

Morphological character matrix for phylogenetic reconstruction

Figures S1 to S6

**Description.**

Body elongated, approximately 50.3 mm in length from the anterior margin of the tooth to the posterior margin of the abdomen (figure 1, S1). Abdominal segments I–IX exhibiting nearly parallel sides, with segment X narrowing slightly towards the posterior margin (figure 1, S1). Body relatively flexible, with the mandibles, lacinial apices, and cerci apparently sclerotized (figure 1, S1).

Head nearly triangular, approximately 0.09× body length, covered with setae dorsally; frontal ridge U-shaped, with two longitudinal sclerotized bands internally; coronal ridge extremely reduced to the posterior margin of the capsule; the ventral side of head bearing fewer setae (figure 2A, B, D). Outline of the anterior external margin of the head capsule not visible due to poor preservation; mentum and admentum positioned between the anterior third and anterior fifth of the head, anterior margin approximately extending to the posterior end of the mandible (figure 2D, S2D‒F). Antennae short, approximately 3.4 mm in length, constituting 0.06× the body length, consisting of 22 visible antennomeres; antennomeres telescopic, with the basal ones not visible, and narrowing toward the tip from the distal 8^th^ antennomere; setae and trichobothria on the antennomeres obscured due to preservation bias (figure 2B). Eyes completely absent (figure 2A, B).

The right mandible robust, with three prominent teeth along the inner margin of the anterior two-thirds; the outer margin arched; the first two teeth exhibiting nearly equal size, being twice as large as the third; each tooth bearing a sclerotized ridge dorsally; condyle spherical (figure 2A, B, S2A‒F). The left mandible partially preserved, bearing three nearly equal-sized teeth (figure S2D‒F). Lacinia approximately one-third the length of the head, positioned longitudinally posterior to the mandible, lacking pectinated laminae; the basal part of lacinia widest, rapidly narrowing at the basal one-fifth, and tapering gradually to a pointed, and slightly inward-curved apex; stipes rectangular, while the cardo oval and composed of two circular joints (figure 2A, B, S2A‒C). Galea poorly preserved, but appearing to have two segments (figure 2B, D, S2B, C, E, F). Maxillary palps consist of four segments, with some setae visible (figure 2A, B, S2A‒C). Labial palps composed of five segments; basal segment longest, progressively shortening and narrowing towards distal segment (figure 2D, S2D‒F). Pharynx located posterior to the mandible, at the center of the head, with distinct circular musculature (figure 2A, B, D, S2A‒F).

The thoracic segments nearly equal in length, gradually increasing in width from segment I to III; both the basal and ventral sides bearing some setae, with a few macrosetae on the basal side, although their distribution pattern remaining unclear; details of the ventral thoracic structures not discernible (figure 1, S1). Legs typical, densely covered with setae; forelegs and midlegs incomplete due to poor preservation; hindleg measuring approximately 9.5 mm, extending to the anterior margin of abdominal segment IV; coxa, trochanter, and femur of hindleg robust, with the coxa nearly as long as the trochanter; tibia approximately twice the length of the coxa; tarsus unsegmented, and the pretarsus equipped with two claws, though the medial unguiculus not visible (figure 2E, S1, S2H, I).

Abdomen telescoped, with prescuta not visible (figure 1, S1). Abdominal segments I and II are the shortest, with nearly equal lengths, followed by segments that gradually lengthen until segment VI; segments VII and VIII progressively shorter, while segment IX becoming longest; segment X 0.63× as long as wide (figure 1, S1). Urotergites bearing setae and macrosetae, although the distribution pattern of the macrosetae unclear (figure 1, S1). Urotergites I–VII slightly sclerotized; urotergites VIII–X not distinctly sclerotized. Urosternites possessing some setae, with styli on segments I–VII; urosternites VIII–IX narrower, and the basal portion of urosternite X fused with the pleurite (figure 1, S1). Median glandular organ and lateral subcoxal organ on urosternite I not visible (figure 1, S1). Styli slender, conical, and tapering towards the apex (figure 2F, F’). A pair of genital appendages positioned between abdominal segments VIII and IX, with each appendage consisting of at least three segments, tapering from the base toward the distal end (figure 2H, H’). A portion of the digestive tract (probably part of hindgut) visible from the middle of abdominal segment IX to the middle of the abdominal segment X (figure 1).

Cerci equal; length ca. 3.15 mm, shorter than segment X (ca. 3.80 mm); widest at base, tapering gradually to pointed, inward-curved apices; internal margin smooth, sharp, without teeth or denticles, biseriate, with a central groove (figure 2H, S2K, L).

**Remarks.**

The giant Jurassic *Sinojapyx giganteus* displays many diagnostic features of Diplura, including a pair of multi-segmented antennae (figure 2A, B), three thoracic segments with each bearing a pair of five-segmented legs (figure 1, 2E, S2G‒I), 10 abdominal segments with a pair of conical styli on urosterites I to VII (figure 1, 2F, F’, G), and a pair of cerci (figure 2H) [1]. The specialized pincer-like morphology of cerci suggests its close relationship with Japygoidea. In contrast to extant japygoids, *S*. *giganteus* exhibit many distinctive features. The head of *S. giganteus* has a subtriangular outline (figure 2A, B), unlike the trapezoidal heads of extant japygoids [1‒3]. Recent japygoids possess slender mandibles with several teeth at the distal [4,5]. However, *S. giganteus* has a well-developed mandible featuring three robust teeth occupying the anterior two-thirds (figure 2A, S2A‒C). Both extant Projapygoidea and Japygoidea have a specialized lacinia characterized by a curved, acute distal lobe with pectinate laminae along the inner margin (figure 2C) [4,6‒11]. Remarkably, the lacinia of *S. giganteus* is only slightly curved and lacks pectinate laminae (figure 2A, B, S2A‒C). Similarly, the lacinia of Campodeoidea appears to be less strongly curved than that of Japygoidea [12,13], but it bears tongue-shaped, smooth laminae along the inner margin [13].

In extant Campodeoidea and Projapygoidea, maxillary palps are unsegmented [9,10,12], whereas Japygoidea display a maximum of three segments, as seen in heterojapygids [5]. By contrast, the maxillary palps of *S. giganteus* comprises at least four segments (figure 2A, B, S2A‒C). Labial palps, which vary significantly across dipluran lineages, range from fully developed to reduced or entirely absent [1]. In most diplurans, labial palps typically consist of at most a single segment (figure 2C), but *S. giganteus* possesses labial palps with five segments (figure 2D, S2D‒F). Paleozoic hexapods, such as the Archaeognatha *Dasyleptus* species, also exhibit more segments in their palps and abdominal appendages than their modern counterparts [14].

Additionally, modern japygoids typically display narrowed and highly sclerotized abdominal segments VIII‒X [15,16]. In contrast, these segments in *S. giganteus* do not show a more pronounced degree of sclerotization compared to the preceding abdominal segments (figure 1). Another distinguishing characteristic is the unreduced abdominal segment IX (figure 1, 2H), which resembles that of extant campodeoids and projapygoids; in contrast, this segment has been reduced to the shortest abdominal segment in japygoids since at least the Cretaceous [17]. Meanwhile, the sternite of segment X in *S. giganteus* is partially fused with the pleurite (figure 2H), unlike the complete fusion seen in modern japygoids. The genital papilla is located between abdominal segments VIII and IX in extant diplurans [15,18], where a pair of unsegmented genital appendages are present on the papilla of male japygoids [16,18,19]. In *S. giganteus*, the genital appendages resemble the single pair of valved structures in males of *Leipojapyx* (Dinjapygidae), whereas females have two pairs of valves [8]. Nonetheless, in no case are they segmented as observed in the fossil. Notably, gonopods are also well-developed in both sexes of the earliest-diverging insects, Archaeognatha, and exhibit a multi-segmented structure [20]. The cerci have evolved specialized features regarding relative length and width, denticulation along the inner margin, and curvature in modern japygoids [21]. However, *S. giganteus* displays simpler cercal structures, characterized by equal cerci and a smooth inner margin (figure 2H, S2L).

**References**

1. Sendra A. 2015 Orden Diplura. *Revista IDE@ - SEA* **35**, 1–11.
2. González RH. 1964 Japygoidea de Sud America, 6: revision de la familia Dinjapygdae. *Acta* *Zool*. *Lilloana* **20**, 113–128.
3. García-Gómez A. 2010 Nueva especie de *Ctenjapyx* (Diplura: Dicellurata: Evalljapygidae) de Chamela, Jalisco, México. *Boletín de la* *S*.E.*A*. **47**, 131–134.
4. Paclt J. 1957 Diplura. In *Genera* *Insectorum* (ed P Wytsman), pp. 1–123. Brussels, Belgium: Louis Desmet-Verteneuil.
5. Blanke A, Machida R. 2016 The homology of cephalic muscles and endoskeletal elements between Diplura and Ectognatha (Insecta). *Org*. *Divers*. *Evol*. **16**, 241–257. (doi:10.1007/s13127-015-0251-5)
6. Verhoeff KW. 1904 Zur vergleichenden Morphologie und Systematik der Japygiden, zugleich zweiter Aufsatz über den Thorax der Insekten. *Arch*. *Naturgesch*. **70**, 63–114.
7. Silvestri F. 1933 Sulle appendici del capo degli Japygidae (Thysanura Entotropha) e rispettivo confronto con quelle dei Chilopodi dei Diplopodi e dei Crostacei. *5th Congr*. *Int*. *Entomol*. 329–343.
8. Smith LM. 1959 Japygidae of South America I. New genus and species of the Danjapyginae (Diplura). *Proc*. *Entomol*. *Soc*. *Wash*. **61**, 27–32.
9. Smith LM. 1960 The family Projapygidae and Anajapygidae (Diplura) in North America. *Ann*. *Entomol*. *Soc*. *Am*. **53**, 575–583. (doi:10.1093/aesa/53.5.575)
10. Rusek J. 1982 *Octostigma* *herbivora* n. gen. & sp. (Diplura: Projapygoidea: Octostigmatidae n. fam.) injuring plant roots in the Tonga Islands. *N*. *Z*. *J*. *Zool*. **9**, 25–32. (doi:10.1080/03014223.1982.10423833)
11. Muegge MA. 2004 Description of *Evalljapyx* *limpia*, n. sp. (Diplura: Japygidae) from the Davis mountains of western Texas. *Proc*. *Entomol*. *Soc*. *Wash*. **106**, 923–927.
12. Silvestri F. 1948 Distribuzione geografica di alcuni piccoli Artropodi: I. *Procampodea* *brevicauda* Silv. (Insecta Diplura). *Ital*. *J*. *Zool*. **15**, 19–24. (doi:10.1080/11250004809440054)
13. Christian E, Bauer T. 2007 Food acquisition and processing in Central European Diplura (Hexapoda). In *Contributions to Soil Zoology in Central Europe II* (eds K Tajovský, J Schlaghamerský, V Pižl), pp. 15–18. České Budějovice, Czech Republic: Proceedings of the 8th Central European Workshop on Soil Zoology.
14. Kukalová-Peck J. 1987 New Carboniferous Diplura, *Monura*, and Thysanura, the hexapod ground plan, and the role of thoracic side lobes in the origin of wings (Insecta). *Can*. *J*. *Zool*. **65**, 2327–2345. (doi:10.1139/z87-352)
15. Denis JR. 1949 Ordre des Diploures. In *Traité de Zoologie IX* (ed PP Grassé), pp. 160–185. Paris, France: Masson.
16. Pagés J. 1989 Sclérites et appendices de l'abdomen des Diploures (Insecta, Apterygota). *Archs*. *Sci*. *Genève* **42**, 509–551.
17. Wilson HM, Martill DM. 2001 A new japygid dipluran from the lower Cretaceous of Brasil. *Palaeontology* **44**, 1025–1031. (doi:10.1111/1475-4983.00213)
18. Koch M. 1997 Monophyly and phylogenetic position of the Diplura (Hexapoda). *Pedobiologia* **41**, 9–12. (doi:10.1016/S0031-4056(24)02965-2)
19. Silvestri F. 1948 Contributo alla conoscenza degli Japygidae (Insecta Diplura). *Società italiana delle scienze detta dei XL* **27**, 1–115.
20. Sturm H, Machida R. 2001 Archaeognatha. In *Handbook of Zoology, Volume 4, Arthropoda: Insecta* (eds NP Kristensen, RG Beutel), pp. 1–213. Berlin, Germany; New York, USA: Walter de Gruyter.
21. Sendra A, Sánchez-García A, Hoch H, Jiménez-Valverde A, Selfa J, Moutaouakil S, Du Preez G, Millar I, Lopes Ferreira R. 2023 Life in darkness: an overview of cave-adapted japygids (Hexapoda, Diplura). *Eur*. *J*. *Taxon*. **894**, 1–54. (doi:10.5852/ejt.2023.894.2287)

**Morphological characters and character states used in phylogenetic analyses**

1. Mandible prostheca: absent (0) present (1)

2. Lacinial lamellae: tongue-shaped and smooth (0) pectinate (1)

3. Lacinia apices: with two teeth (0) pointed (1) bifurcated (2)

4. Maxillary palps: absent (0) present (1)

5. Palpiform process: absent (0) present (1)

6. Cupuliform organ: absent (0) present (1)

7. Number of thorax spiracles: two pairs (0) three pairs (1) four pairs (2)

8. Styli on urosternite I: absent (0) present (1)

9. Abdominal spiracles: absent (0) present (1)

10. Abdominal segment IX: subequal to or longer than segment VIII (0) / distinctly shortest (1)

11. Body cuticle sclerotization: absent or very weak (0) present and distinct (1)

12. Abdominal segment X: not highly sclerotized (0) highly sclerotized (1)

13. Cerci length: long (>0.5× body) (0) short (<0.5× body) (1)

14. Cerci segmentation: multisegmented (0) unsegmented (1)

15. Apical glandular pore on cerci: absent (0) present (1)

16. Inner margin of pincer-like cerci: smooth (0) with teeth or denticles (1)

17. Ovary structure: consisting of two simple sacs (0) divided into two oviducts (1)

**Morphological character matrix for phylogenetic reconstruction**

|  | 1 | 2 | 3 | 4 | 5 | 6 | 7 | 8 | 9 | 10 | 11 | 12 | 13 | 14 | 15 | 16 | 17 |
| --- | --- | --- | --- | --- | --- | --- | --- | --- | --- | --- | --- | --- | --- | --- | --- | --- | --- |
| Campodeidae | 1 | 0 | 0 | 0 | 1 | 1 | 1 | 0 | 0 | 0 | 0 | - | 0 | 0 | 0 | - | 0 |
| Procampodeidae | 1 | 0 | ? | 0 | 1 | 1 | 0 | 0 | 0 | 0 | 0 | - | 0 | 0 | 0 | - | 0 |
| Projapygidae | 1 | 1 | 1 | 1 | 1 | 0 | 1 | 1 | 1 | 0 | 0 | - | 1 | 0 | 1 | - | 1 |
| Anajapygidae | 1 | 1 | 2 | 1 | 1 | 0 | 0 | 1 | 1 | 0 | 0 | - | 1 | 0 | 1 | - | 1 |
| Octostigmatidae | 1 | 1 | 2 | 1 | 1 | 0 | 2 | 1 | 1 | 0 | 0 | - | 1 | 0 | 1 | - | 1 |
| Dinjapygidae | 0 | 1 | 1 | 1 | 0 | 0 | 0 | 1 | 1 | 1 | 1 | 1 | 1 | 1 | - | 1 | 1 |
| Heterojapygidae | 0 | 1 | 1 | 1 | 0 | 0 | 2 | 1 | 1 | 1 | 1 | 1 | 1 | 1 | - | 1 | 1 |
| Evalljapygidae | 0 | 1 | 1 | 1 | 0 | 0 | ? | 1 | ? | 1 | 1 | 1 | 1 | 1 | - | 1 | 1 |
| Japygidae | 0 | 1 | 1 | 1 | 0 | 0 | 2 | 1 | 1 | 1 | 1 | 1 | 1 | 1 | - | 1 | 1 |
| Parajapygidae | 0 | 1 | 1 | 1 | 0 | 0 | 0 | 1 | 1 | 1 | 1 | 1 | 1 | 1 | - | 1 | 1 |
| *Sinojapyx giganteus* | ? | - | 1 | 1 | ? | 0 | ? | 1 | ? | 0 | 1 | 0 | 1 | 1 | - | 0 | ? |
| *Ferrojapyx vivax* | ? | ? | ? | 1 | ? | 0 | ? | ? | ? | 1 | 1 | 1 | 1 | 1 | - | 0 | ? |

**Figure S1.** General habitus of *Sinojapyx giganteus* in ethanol. (A) counterpart. (B) part. Scale bar = 0.5 cm.

 **Figure S2.** Details and corresponding line diagrams of *Sinojapyx giganteus*, with a comparison to the extant *Heterojapyx* sp. (A–C) Mouthparts on the part. (D–F) Mouthparts on the counterpart. (G) The scanning electron micrograph of the left foreleg of *Heterojapyx*. (H–I) Right hindleg of *S. giganteus.* (J) Coronal view of abdominal segment X and cerci of *Heterojapyx*, captured through micro-CT scanning. (K–L) Abdominal segments IX–X with pincer-like cerci. Abbreviations: ad, admentum; cl, claw; co, coxa; fu, femur; ga, galea; la, lacinia; lp, labial palps; ma, mandible; me, mentum; mp, maxillary palps; mu, muscles; ph, pharynx; ta, tarsus; ti, tibia; tr, trochanter. Scale bars: A–F, 500 µm; G, 250 µm; H–J, 1000 µm; L, K, 2000 µm.

A

B

**Figure S3.** Strict consensus tree. (A) Result of the 12-taxa matrix (including *Sinojapyx giganteus*). Consistency Index (CI) = 0.826, Retention Index (RI) = 0.882, Tree Length (TL) = 23. (B) Result of the 11-taxa matrix (excluding *Sinojapyx giganteus*). CI = 0.818, RI = 0.875, TL = 22.

C

B

**Figure S4.** Majority rule consensus tree. (A) Result of the 12-taxa matrix (including *Sinojapyx giganteus*). CI = 0.826, RI = 0.882, TL = 23. (B) Result of the 11-taxa matrix (excluding *Sinojapyx giganteus*). CI = 0.818, RI = 0.875, TL = 22.

A

B

**Figure S5.** Semi-strict consensus tree. (A) Result of the 12-taxa matrix (including *Sinojapyx giganteus*). CI = 0.864, RI = 0.912, TL = 22. (B) Result of the 11-taxa matrix (excluding *Sinojapyx giganteus*). CI = 0.857, RI = 0.906, TL = 21.

**Figure S6.** A Lepidocampinae species, NIGP205817. (A) Ventral view of the specimen. (B) Claw on the left midleg. (C) Scales on the abdomen. (D) Scales on the thorax. Scale bars: A, 500 µm; B, 10 µm; C, D, 20µm.
